# A Proof of Concept: Are Detection Dogs a Useful Tool to Verify Potential Biomarkers for Lung Cancer?

**DOI:** 10.3389/fvets.2018.00052

**Published:** 2018-03-14

**Authors:** Carola Fischer-Tenhagen, Dorothea Johnen, Irene Nehls, Roland Becker

**Affiliations:** ^1^Clinic of Animal Reproduction, Freie Universität Berlin, Berlin, Germany; ^2^Bundesanstalt für Materialforschung und -prüfung (BAM), Berlin, Germany

**Keywords:** detection dog, breath, sampling, biomarkers, synthetic air, lung cancer

## Abstract

Early and reliable diagnostic test is essential for effective therapy of lung cancer. Volatile organic compounds that are characteristic for cancer could serve as valuable biomarkers in cancer diagnosis. Both trace analytical and detection dog approaches give some evidence for the existence of such biomarkers. In this proof of concept, study dogs and trace analysis were implemented in combination to gain more information concerning cancer biomarkers. Two dogs were trained to distinguish between absorbed breath samples of lung cancer patients and healthy persons and succeeded with correct identification of patients with 9/9 and 8/9 and correct negative indications from of 8/10 and 4/10 samples from healthy individuals. A recent observational study found that breath samples from lung cancer patients showed an increase in 1-butanol, 2-butanone, 2-pentanone, and hexanal. Synthetic air samples were therefore fortified with these compounds and adsorbed to a fleece. Tested against breath samples from healthy probands, on presentation to the dogs these synthetic samples provoked an indication in three out of four samples. We were able to demonstrate that a combination of the natural nose of a dog and a trace analytic technique can be a valuable concept in the search for cancer biomarkers.

## Introduction

Lung cancer is one of the most frequent cancer forms in Europe. In 2012, it was the leading type of cancer for males and the third most frequent type for females, causing 353.000 deaths in the 745 billion Europeans (1). Early and accurate diagnostic test is essential for improving the 5-year survival rate. There is science-based evidence that dogs are able to identify cancer specific odors in breath, blood, and urine samples of cancer patients (2–4). Although some studies reported promising sensitivities (71–99%) and specificities (78–98%) (5–8), other studies described discouraging test characteristics (sensitivities: 3–71% and specificities: 8–53%) and discussed this approach more critically (4, 9–12). These controversial results led to the question whether there are specific volatile organic compounds (VOCs) that are characteristic for a certain type of cancer cell or for metabolic processes in patients suffering from cancer.

Filipiak et al. (13) found that lung cancer cell lines and non-pathogenic cells cultured *in vitro* showed significant differences in the headspace in gas chromatography-mass spectrometry. However, in a study by Schallschmidt et al. (14) dogs and honey bees failed to discriminate between air from *in vitro* cultured lung cancer cells and cell-free culture medium. Other approaches for biomarker identification used *in vivo* cancer models (15) and resected tumor tissue instead of cell cultures (7, 16, 17) which were analyzed with respect to differences in VOC profiles of pathogenic and non-pathogenic samples.

Volatile organic compounds in breath samples of patients suffering from lung cancer have additionally been investigated using gas chromatography. Aliphatic aldehydes were among the compounds repeatedly suggested to display increased levels in exhaled breath of patients (18–20). In addition, aliphatic aldehydes have been detected in urine (21) and blood (22) of lung cancer patients. Butanol (20, 23) and volatile 2-oxoalkanes (7, 20, 24) in breath have also repeatedly been associated with lung cancer.

The only attempt to combine detection dogs with instrumental VOC analysis to distinguish between healthy and diseased people was reported by Buszewski et al. (7). Dogs’ indication of breath odor adsorbed to polypropylene tubes were compared with gas chromatography/mass selective detection data of VOC in breath samples taken with Tedlar bags from the same individuals.

Nevertheless, so far no single VOC or set of VOCs has reached clinical relevance for reliable disease recognition with sufficient sensitivity and specificity.

The objectives of this study were

(i) to set up a system that allows sampling and handling of breath for detection dog training, testing in an unbiased and reproducible way and the ability to spike samples with potential biomarkers;
(ii) to test if dogs can be trained using this set-up to distinguish between breath of lung cancer patients and healthy controls;
(iii) to test how these dogs react to synthetic air samples with potential volatile biomarkers observed in breath. These compounds were 1-butanol, 2-butanone, 2-pentanone, and hexanal, which have previously been associated with lung cancer (20).

## Materials and Methods

### Material and Technological Developments

**Figure 1:**
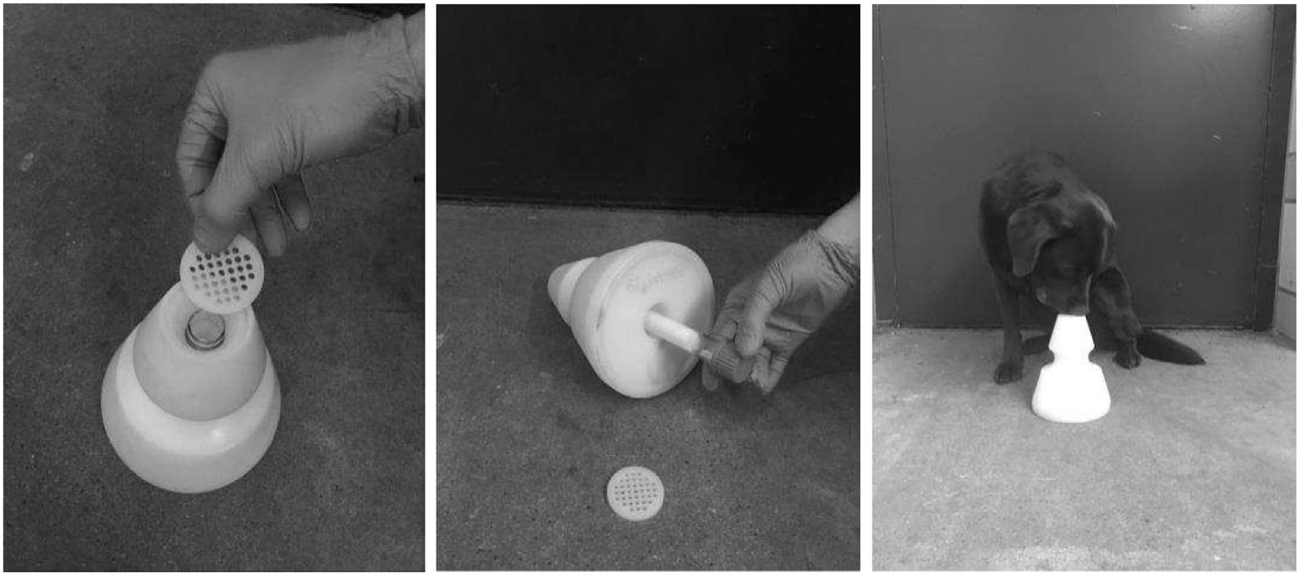
Polyethylene cone for presenting glass tubes to dogs in training and testing. Left picture: perforated plate changed after every contact of a dog’s nose; middle picture: glass tube with air sample attached to fleece is inserted into cone. Right picture: dog indicates a cone with a sitting response.

The main innovations were the improved fleece tubes for breath sampling and the cone-shaped sample-holder (Figure 1, patent DE 10 2013 109 901.7) that allowed placing and changing the fleece tube easily for detection dog training and breath sample testing. The design of the fleece tubes and the sample-holders enabled reproducible test runs, efficient cleaning of devices between runs and avoided contaminations of memory effects.

### Genuine Breath Samples

Probands (patients *n* = 30 and healthy controls *n* = 30) participating in the study were enrolled in the cooperating clinic (Evangelische Lungenklinik, Berlin) following a jointly developed standard operating procedure for breath sampling, documentation of medical status, and ensuring patient anonymity. Breath samples of patients were taken on the first visit in the lung clinic for diagnosis, patients were not on any cancer treatment. Control persons were matched concerning age, sex and smoking habits. Patients and controls were not fasted prior to testing. The involvement of probands was approved by the Ethics Committee of the Charité, Berlin, Germany, and registered as clinical trial with proof-of-concept (EA1/207/13). An absorber technique using polypropylene fleece, developed on the basis of findings by Ehmann et al. (25) was chosen for breath sampling. The sampling tubes dedicated for dog training and testing consisted of a glass tube (length: 150 mm, inner diameter: 18 mm) with GL25 sockets on both sides (Gaßner Glastechnik, Munich, Germany). Each tube was filled with two 70 mm × 43 mm polypropylene fleeces (Asota GmbH, Linz, Austria) (Figure 1). One was hydrophilically (Asota^®^ olefin hydrophilic) and the other was hydrophobically (Asota^®^ olefin hydrophob) modified. Tubes were closed with silicone septa for GL25 sockets (neoLab Migge Laborbedarf-Vertriebs GmbH, Heidelberg, Germany) and with polybutylene-terephthalate screw caps (Bohlender GmbH, Grünsfeld, Germany) (26, 27). During each breath sampling the volume of the airflow was monitored with a spirometer (Ganshorn Medizin Medizin Electronic GmbH, Niederlauer, Germany). Each patient donated two fleece tube samples with a resting time of 10 min in between. Samples were stored refrigerated at 8°C until use. For dog training, 21 patient samples and 20 controls samples were used, 9 novel patient samples and 10 novel samples of controls were introduced for testing.

### Synthetic Air Samples

Humidified synthetic air (80% N_2_, 20% O_2_; relative humidity: 84–89%) in a 1 L glass beaker with lateral septum as described in detail elsewhere (20) was spiked with 10 µL of a demineralized water solution containing 1-butanol (10.4 µg/L), 2-butanone (9.7 µg/L), 2-pentanone (3.2 µg/L), and hexanal (5.4 µg/L). The concentrations of the four compounds were chosen such that their concentrations in the glass bulb after injection through the septum were equal to the respective medians found in the breath of lung cancer patients in an observational study (20). After an equilibration period of 30 min, the air was transferred onto a fleece tube (see above) through a glass tube by means of an argon flow (160 mL/min for 30 min). Fleece tubes were closed tight and stored refrigerated at 8°C until use. A total of four fleece tubes loaded with spiked synthetic air were prepared.

### Dog Training

All training was performed at the dog training and testing lab, Freie Universität Berlin. Four privately owned dogs were trained in this study, one 5-year-old spayed Labrador bitch, a 3-year-old intact female poodle, a 7-year-old female intact Dachshund, and an 8-year-old spayed Labrador Mix bitch. Selection was by convenience. Inclusion criteria were: dogs had to be clinically healthy, regularly available for training and familiar with training and testing of odor discrimination procedures (27).

In accordance with the European legislation (Dir. 2010/63/EU), no animal was exposed to harmful conditions throughout this study. During the study the dogs lived in their familiar home. The trainers had contributed to earlier odor detection studies with dogs (14, 28, 29). Training was conducted between June and December 2015–2016. The Labrador Mix and Dachshund did not make a considerable training progress in discriminating breath samples and were excluded from training after 3 months. Numbers of training days for the Labrador and the poodle were 73 and 82 days, respectively, with training trials (decision on a presented odor sample) ranging from 5 to 20 (average 15) per training day. Training took place only once a week, dogs were not trained at the same time or same day.

Training methods were based on positive reinforcement using a clicker as a secondary reinforcement and small food treats as reward and dogs were off leash during training and testing. Every dog was rewarded with its favorite food. In case of a wrong indication, a reward was not given and the dog had to pause for at least 1 min before repeating the trial. The dogs were familiar with the sound of the clicker as a predictor for food. A minimum of 80% correct indications were required in order to progress to the next training step. In brief, the training approaches included following steps:

In the first step, a cone with a fleece tube sampled from a lung cancer patient as positive sample was presented to the dog, and the dog was immediately rewarded for sniffing the holder. The cone was standing on the floor approximately 1 m away from the dog. Then, the dog was trained to indicate the cone by standing still and pointing or sitting next to it. In step two, a second, empty cone was introduced and placed approximately 50 cm away from the positive cone. The dog was required to identify and indicate the cone with the positive sample. In the third step, the second cone was loaded with a negative fleece tube sample (sampled from a healthy person) and the number of cones was increased to four (one positive, three negatives). Thus, the dog had to make a one-out-of-four decision (25% chance). After the dogs had performed a minimum of 80% correct indications, training was conducted in a double-blind manner. The dog handlers were not aware of the position of the positive sample and the experimenter was in a cubicle separated from the test room by a non-transparent curtain.

In the final training phase, number of positive cones per trial could vary from 0 to 4. When there was no positive in the trial, the dog was rewarded for returning to the handler after sniffing at all cones.

The perforated plate on top of a cone (positive or negative) was replaced whenever a dog’s nose had contact to it. Plates were cleaned in an ultrasonic bath for 90 s. The cones were wiped with a wet cloth to minimize the risk of scent contamination. Fleece tube samples were used multiple times and stored refrigerated at 8°C in between training days.

### Dog Testing

Dog testing took place in the same room as dog training. Two tests were performed in the scope of this study. The first test was to evaluate ability of the dogs’ to distinguish between sampled breath from cancer patients and healthy controls. In the second test, we included synthetic air samples spiked with 1-butanol, 2-butanone, 2-pentanone, and hexanal. No sample used in training was used for the test.

For the first test, we used 9 cancer positive breath samples and 10 samples from healthy controls. The samples were used up to three times. Each dog was presented with a total of 40 samples. We documented the reaction of the dogs at the first contact of the sample to avoid studying a memory effect.

The samples were presented in trials. Each trial consisted of 2–4 cones presented to the dog. The cones were placed 40 cm apart from each other and at a distance of 2 m from the dog’s starting point. The number of cones with positive samples in one trial was random and could vary from 0 to 4.

Randomization both for samples within a trial and over all trials occurred using the random number function of Excel (Microsoft Office, Microsoft Cooperation, Redmond, WA, USA).

A positive indication varied between dogs and consisted of the dog in question either sitting down at the cone or standing still, nose pointing at the cone, for a minimum of 3 s. After a negative indication the dog moved on to the next cone. The handler announced each indication to the experimenter.

In case of a correct positive indication, the dog was rewarded and the trial was finished. Cones the dogs had not sniffed remained in the lineup for the next trial, to make sure that dogs had to make decisions on every single cone. If the trial had no positive sample the dog was rewarded when it returned to the handler after sniffing all cones. Correct negative or false negative decisions were documented. Dog, handler, and any other person in the room were blinded to the position of the sample to avoid hidden clues.

In the second test, we wanted to observe the reaction of the dogs when they were presented with synthetic air samples. Therefore, 4 spiked synthetic air samples served as positive samples and all 10 healthy controls were included in the test. We presented 40 samples to the dogs. All synthetic air samples of the same composition, but different preparation days.

### Statistical Data Analysis

The experimental set-up for breath samples with the random presentation of positive or negative samples led to the identification of any sample as true positive, true negative, false positive, or false negative. In the second test, only breath samples from healthy probands served as controls but no synthetic control samples. Therefore, true negative rate was not calculated.

## Results

In the first test, the Labrador had a correct identification rate at first presentation of nine out of nine and the Poodle of eight out of nine. True negative rate was 8 out of 10 for the Labrador and 4 out of 10 for the poodle. Results are summarized in Tables 1 and 2.

**Table 1:** Indication (+ = correct; − = false) of dog at first contact with breath sample of patient suffering from lung cancer.

Sample ID	1024	1025	1026	5074	5075	5076	5077	5078	5079
Labrador	+	+	+	+	+	+	+	+	+
Poodle	+	+	+	−	+	+	+	+	+

**Table 2:** Indication (+ = correct; − = false) of dog at first contact with breath sample of healthy probands.

Sample ID	1515	1516	1517	1518	1519	7515	7516	7517	7518	7519
Labrador	+	+	+	+	+	+	−	+	+	−
Poodle	−	−	−	+	−	−	−	+	+	+

In the second test with synthetic air sample, both dogs indicated three out four synthetic samples as positive. Results for the first choice of the dog sniffing at the synthetic air samples are summarized in Table 3.

**Table 3:** Indication (+ = correct; − = false) of dog at first contact with synthetic air samples with potential biomarker for lung cancer.

Sample ID	1_DOT	2_DOT	3_DOT	4_DOT
Labrador	+	−	+	+
Poodle	+	+	+	−

## Discussion

In this study, two dogs were successfully trained to distinguish between breath samples from cancer patients and healthy controls. During the course of the study, four adult dogs undertook training. Due to insufficient training progress, two dogs were excluded after 3 months of training.

With regard to similar studies, Elliker et al. (12) reported that 7 out of 10 dogs in training were unable to reach the final stages of training. In the study of Gordon et al. (10), only 4 out of 10 dogs learnt to detect breast or prostate cancer in urine of human patients.

Due to the low number of dogs included in detection dog studies, it is difficult to prove an influence of the individual dog on the result statistically. We assume that some dogs have a higher ability to be trained for cancer detection than others. Further research is needed to identify selection criteria for the best possible cancer detection dog. Number of dogs in studies on cancer detection with dogs varies from 1 to 10 (28).

Unfortunately, only two dogs progressed to the final stage of training and could thus be included in the testing. While this is clearly an insufficient sample size, both dogs were able to indicate synthetic air samples as positive for cancer, which provides some proof of concept.

Our training duration of 16 months was long in comparison to shorter training periods described by other authors of 3 weeks (5), 7 months (9), or 12 months (6). Frequency of training in our study was once a week, which may have led to the longer total training period required. Number of sample of probands was limited, so we had to use same samples for multiple training sessions. This bears the danger of teaching dogs the individual odor of these probands instead of the specific odor of cancer (4). For the test, we used 19 samples (9 positives and 10 negatives) of probands the dogs had no contact with before. For this reason, we only documented the reaction of the dogs at first contact with the samples.

Ability for distinguishing the probands is within the range of previous studies conducted on lung cancer detection by detection dogs. With regard to all articles published so far on canine detection of lung cancer in humans on the basis of breath odor, the mean sensitivity was 78%, whereas the mean specificity was 71.5% (3). The results of the studies differ substantially. Whilst McCulloch et al. (5) found a sensitivity and specificity of 99% in detection of positive breath samples on lung cancer patients, the study by Amundsen et al. (11) revealed a mere 55.6% sensitivity and 8.3% specificity for small cell lung cancer. For a review of studies on lung cancer detection by detection dogs refer to Pirrone and Albertini (3).

The ability of the two dogs to discriminate breath samples was deemed satisfactory to continue with the project. The purpose of training the dogs to indicate breath samples of lung cancer patients was to test their response when they sniffed at synthetic air samples containing prospective VOCs that have previously been associated with lung cancer (20).

In the test to observe the reaction of dogs to synthetic air samples, both dogs indicated three out of four samples as cancer positive samples. Both dogs assigned the synthetic air samples to the cancer patients’ group. Our results suggest that dogs have potential be used to verify potential biomarkers for lung cancer. As a future perspective, this preliminary finding needs to be reproduced with control samples spiked with substance with no potential for biomarkers. As a next step genuine breath samples should be spiked with potential biomarkers and as controls with other substances found in breath samples not specific for cancer. Potential misleading of the dogs by unknown characteristics of involved genuine or spiked samples have to be ruled out by experimental design.

Lippi and Cervellin (30) suggested that, the “natural nose” of the animal might help to identify candidate biomarkers found by analytic technology. Buszewski et al. (7) found a tendency of greater concentrations of butanal, 2-butanone, ethyl acetate, ethyl benzene, 2-pentanone, 1-propanol, and 2-propanol in the breath of 44 lung cancer patients in comparison to 29 controls. In addition, they trained dogs to distinguish between both groups with a sensitivity of 82.2% and specificity of 82.4%. They found that ethyl acetate and 2-pentanone correlated positively with the dogs’ positive indications. In a more recent study by this group with 108 lung cancer patients and 121 controls, including healthy probands and patients suffering from other lung diseases, a significant increase of concentrations of 1-propanol, 2-propanol, methyl acetate, hexanal, dimethyl sulfide, and carbon disulfide was found in the group of patients with lung cancer (31). With the help of Chi-squared automatic interaction detection, they were able to show that dimethyl sulfide is the main compound enabling differentiation between two groups: patients with cancer and healthy volunteers. They prepared synthetic samples on the basis of exhaled air of cancer patients. The indication of synthetic samples by the trained dogs was significantly worse (21%) than the indication of breath samples from cancer patients (86% correct positive). Unfortunately, the authors did not describe how the synthetic samples were prepared and which substances were included.

Based on the study by Schallschmidt et al. (20) we included 1-butanol, 2-butanone, 2-pentanone, and hexanal in the synthetic air samples.

Although methods in these studies were different 2-butanone, 2-pentanone, and hexanal were found throughout.

Currently available literature suggests that rather than there being one cancer-specific VOC, a combination of several VOCs display significantly higher concentrations in cancer patients (32). Buszewski et al. (7) stated that the signature odor of cancer that dogs use for differentiation between samples may be related to specific qualitative or quantitative olfactory impressions produced by a mixture of VOCs.

In this study, the two dogs discriminated the synthetic samples against healthy controls. In a more ideal test, it should be assessed if dogs discern between patients samples and synthetic samples, including VOCs potentially specific for cancer and VOCs not suspicious for cancer. This would underline the similarity between synthetic and patient samples.

Further research is warranted to test more combinations of potential biomarkers for lung cancer. We believe that specially trained detection dogs are a useful tool for finding the best possible biomarker for an effective diagnostic system for lung cancer.

## Ethics Statement

The involvement of probands was approved by the Ethics Committee of the Charité, Berlin, Germany and registered as clinical trial with proof-of-concept (EA1/207/13).

## Author Contributions

CF-T and IN were involved in study design, dog training (as well as DJ), and writing the manuscript. RB contributed substantially to the manuscript.

## Conflict of Interest Statement

The authors declare that the research was conducted in the absence of any commercial or financial relationships that could be construed as a potential conflict of interest.
